# ScatLay: utilizing transcriptome-wide noise for identifying and visualizing differentially expressed genes

**DOI:** 10.1038/s41598-020-74564-1

**Published:** 2020-10-15

**Authors:** Thuy Tien Bui, Daniel Lee, Kumar Selvarajoo

**Affiliations:** 1 Singapore Institute of Food and Biotechnology Innovation, Agency for Science, Technology & Research (A*STAR), 61 Biopolis Drive, Singapore, 138673 Singapore; 2 School of Computer Science and Engineering, Nanyang Technological University, 50 Nanyang Avenue, Singapore, 639798 Singapore; 3 Synthetic Biology for Clinical and Technological Innovation (SynCTI), National University of Singapore, 28 Medical Drive, Singapore, 117456 Singapore

**Keywords:** Computational biology and bioinformatics, Systems biology, Genetics, Gene expression

## Abstract

Differential expressed (DE) genes analysis is valuable for understanding comparative transcriptomics between cells, conditions or time evolution. However, the predominant way of identifying DE genes is to use arbitrary threshold fold or expression changes as cutoff. Here, we developed a more objective method, Scatter Overlay or ScatLay, to extract and graphically visualize DE genes across any two samples by utilizing their pair-wise scatter or transcriptome-wide noise, while factoring replicate variabilities. We tested ScatLay for 3 cell types: between time points for *Escherichia coli* aerobiosis and *Saccharomyces cerevisiae* hypoxia, and between untreated and Etomoxir treated *Mus Musculus* embryonic stem cell. As a result, we obtain 1194, 2061 and 2932 DE genes, respectively. Next, we compared these data with two widely used current approaches (DESeq2 and NOISeq) with typical twofold expression changes threshold, and show that ScatLay reveals significantly larger number of DE genes. Hence, our method provides a wider coverage of DE genes, and will likely pave way for finding more novel regulatory genes in future works.

## Introduction

High-throughput and next generation sequencing data analyses have dominated much of biological research in the last decade. The major challenge is to tackle the large dataset into a manageable way for key biological inference. There has been much effort in the development of statistical tools to interpret the data, especially to identify genes that act differently between any two samples, for example, between wild type and mutants or across time for a given stimulus^1–4^.

Till today, the predominant way is to input user defined parameters to select genes for evaluation, such as 2 or threefold differently expressed, sometimes with a given minimum expression value and/or with a statistical null hypothesis (*p* value) criteria^5–7^. These approaches have provided valuable insights into the underlying differential activation mechanisms, nevertheless, to overcome the arbitrarily or biasedly used selection criteria, we require newer methods that provide alternative solutions.

Previously, to reveal how the transcriptional machineries of human and mouse embryonic developmental cells evolve with time, we had quantified and used transcriptome-wide noise (squared coefficient of variation) as a non-parametric metric to observe key differences between the developmental stages^8^. Here, we set a similar approach to track genes that vary or scatter significantly compared with replicate (technical or operator induced) variability.

## Results and discussion

### Transcriptome-wide scatter

**Figure 1:**
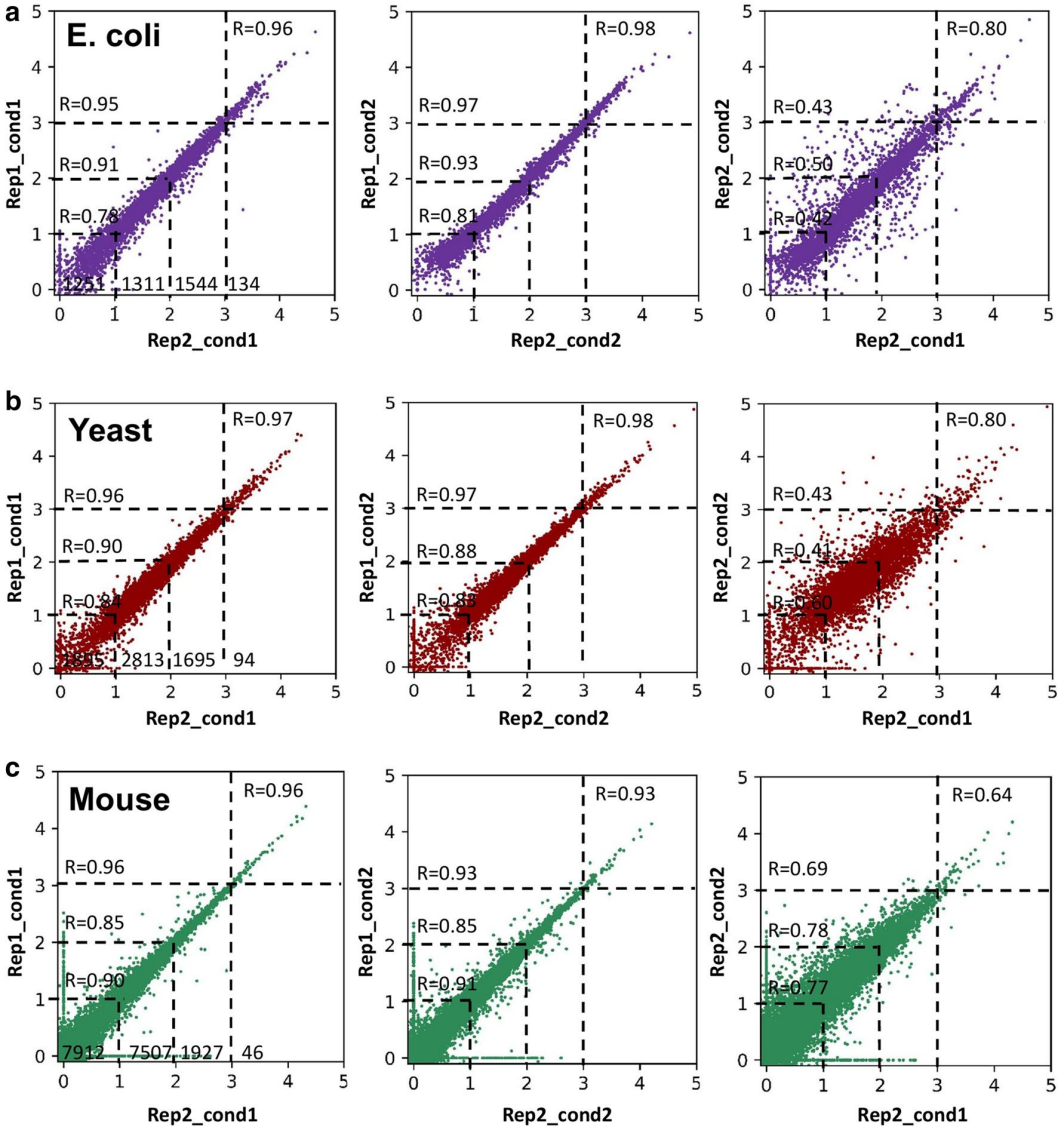
Transcriptome-wide expression scatter. (**a**) *E. coli* (purple), (**b**) *S. cerevisiae* (burgurdy), and (**c**) mouse embryonic stem cell (ESC) derived from blastocysts (green) gene expression scatter between 2 replicates at anchor condition (*t* = 0) for *E. coli* and *S. cerevisiae*, and control condition for mouse ESC, (denoted as cond1, left panel); between 2 replicates at target condition for *E. coli* (*t* = 10 min) and *S. cerevisiae* (t = 240 min), and Etomoxir treated condition for mouse ESC, (denoted as cond2, middle panel); between anchor and target condition (right panel).

We obtained RNA-Seq dataset, from the NCBI GEO database, for *Escherichia coli* in aerobiosis, *Saccharomyces cerevisiae* in hypoxia, and *Mus Musculus* embryonic stem cell (ESC) with and without Etomoxir (ETO) treatment (see Materials and Methods). After performing Transcripts Per Kilobase Million or Transcripts Per Million (TPM) normalization of the read counts for all samples, we plotted transcriptome-wide expression scatter between any two replicates and between the anchor time (*t* = 0) and the last time points for both *E. coli* and *S. cerevisiae*, and between untreated and ETO treated mouse ESC cells (Fig. 1a–c).

In all the replicate plots, we observe a scatter that reduces with higher expression values resulting in an increase of Pearson correlation *R* with increasing expressions (Fig. 1a–c, left and middle panels). This is expected as the effect of noise, such as due to technical variability, tend to become less significant for higher expressions^8–10^. Thus, noise is usually a concern for lower gene expressions. We also observe for all cell types that the transcriptome-wide expression scatter widens, with decreasing *R*, when samples are compared from anchor time with other times (Fig. 1a,b, right panels), or untreated with treated (Fig. 1c). This is an indication that certain number of genes are differentially regulated in time or condition; the widening of those gene expressions contributing to the observed scatter.

### Statistical distribution fitting to remove lower expression or noisy genes

It is now known that gene expressions follow certain statistical distributions, such as Pareto (power-law) or lognormal^11–14^. Noting that lowly expressed genes are generally prone to noise^8–10^ (Fig. 1), previously we used the statistical distribution fittings (Materials and Methods) to select genes for further evaluation^13,14^. Here, we adopted the same approach to remove lowly expressed “noisy” genes.

Figure S1 shows the transcriptome-wide distribution of the *E. coli* and *S. cerevisiae* data for all time points, and mouse ESC for control and different treated conditions (Materials and Methods). Comparing with a number of statistical distributions and using Akaike information criterion^15^, we concur that lognormal distribution is the best fit for both *E. coli* and mouse ESC data, while Burr distribution for *S. cerevisiae* (Fig. S1 and Table S1). Using the lower end tail intersection as a threshold, we obtain TPM > 5 for *E. coli*, while TPM > 2 for both *S. cerevisiae* and mouse ESC as the lower expression noise cut-off level. Overall, for subsequent DE gene analysis, we retained 3758, 5330, and 11,787 genes for *E. coli, S. cerevisiae* and mouse ESC data, respectively.

### Quantifying transcriptome-wide scatter as noise

**Figure 2:**
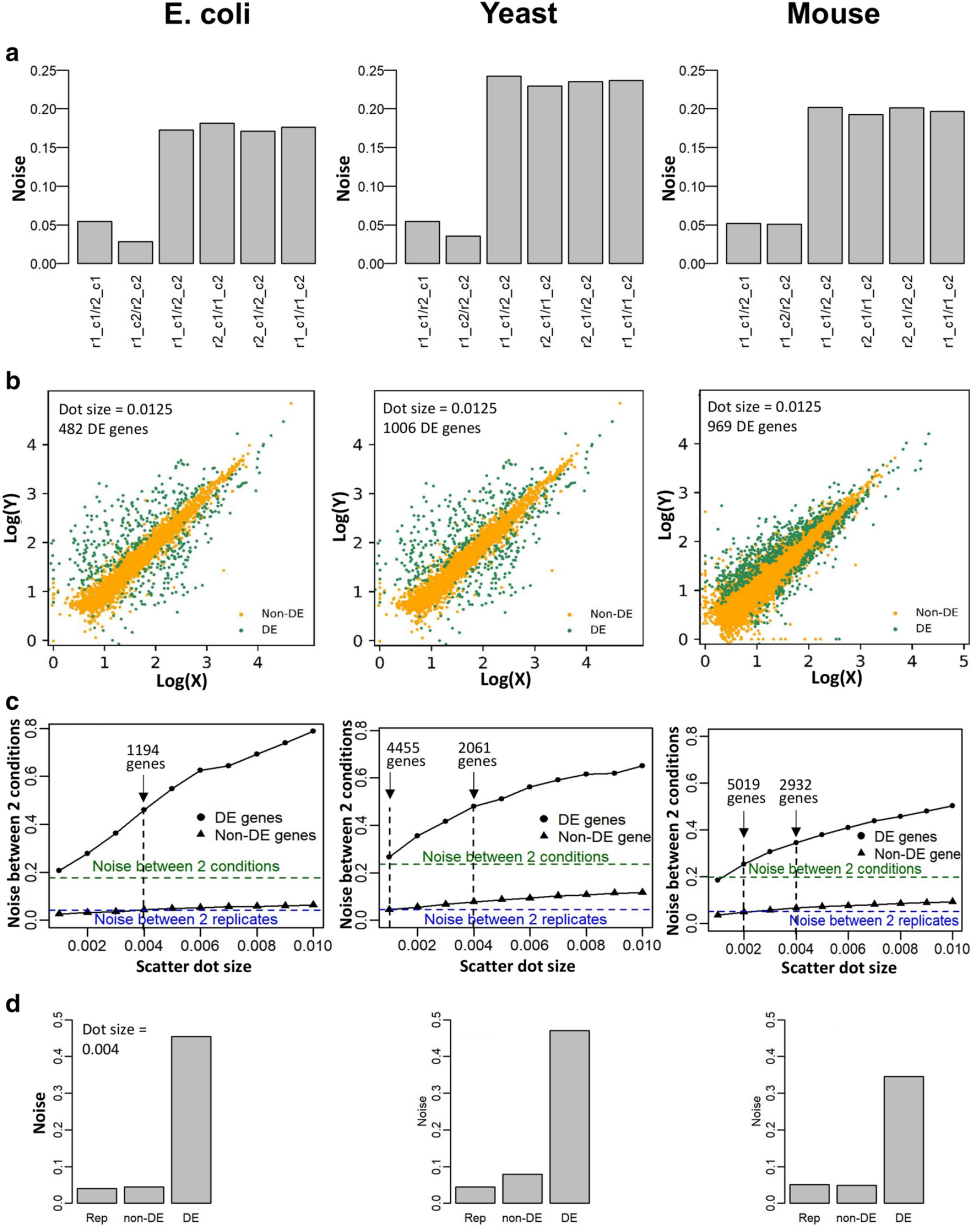
Transcriptome-wide expression noise as an indicator for differential expressions. (**a**) Expression noise between 2 replicates at the same time point (between replicate 1 and replicate 2 of condition 1, denoted as r1_c1/r2_c1 and between replicate 1 and replicate 2 of condition 2, denoted as r1_c2/r2_c2) and between anchor and target conditions in 4 combinations of 2 replicates and 2 conditions, after the removal of lowly expressed genes (Fig. S1). (**b**) Distinction between differentially expressed (DE) genes (green) and non-DE genes (orange) by overlaying expression scatter between 2 conditions and 2 replicates at all conditions in *E. coli* (left panel), *S. cerevisiae* (middle panel), and mouse ESC (right panel). (**c**) Expression noise between anchor and target time points due to DE genes (filled circle) and non-DE genes (filled triangle) with scatter dot size ranging from 0.001 to 0.01 log10(TPM)) in *E. coli* (left panel), *S. cerevisiae* (middle panel), and mouse ESC (right panel). Scatter dot size at 0.004 log10(TPM) for *E. coli*, 0.001 log10(TPM) for *S. cerevisiae*, and 0.002 log10(TPM) for mouse ESC resulted in non-DE gene set whose expression noise between anchor and target time points is comparable to the averaged whole-transcriptome noise between 2 replicates (dashed blue line). The most conservative dot size of E. coli, 0.004 log10(TPM) was applied for all cell types. (**d**) Expression noise at scatter dot size 0.004 for transcriptome-wide replicates (Rep), non-DE and DE genes between 2 different conditions.

To quantify or estimate the transcriptome-wide scatter of the selected genes, we revisit gene expression noise, which is defined by expression variance over square of expression mean (Materials and Methods). Figures 2a and S2 show that transcriptome-wide noise is lower between replicates at any time, compared with the anchor time (*t* = 0) and other time points, or between untreated and treated conditions. The higher noise is mainly due to the differentially expressed genes (DE genes). Note that the level of noise between any two replicates is almost similar (approximately 0.05) for any time points or conditions (Fig. 2a). This indicate the level of noise that one could expect between any two experimental samples due to technical, operator or culture media induced variability^8,16^. Any values beyond this level are most likely a result of the differential transcriptional mechanisms that occurs in time, such as for aerobiosis, hypoxia or between different experimental treatments.

### Identifying differentially expressed genes

The predominant way of identifying DE genes is based on setting arbitrary expression fold change cutoff, e.g. 1.5, 2 or threefold changes^17,18^. Although these methods are generally acceptable for selecting the most highly variable genes, recent works indicate even lowly changing genes play key regulatory roles^19,20^. Hence, a more objective way to identify DE genes can provide a wider spectrum of transcriptional processes at play.

Here, we developed a software with graphic user interface (GUI) to overlay and visualize the transcriptome-wide scatter between any 2 samples (replicates/conditions/time points). The scatters are overlaid over each other, and when the expression of any element (gene) of the dataset become overlapping, its original color (e.g. green) will change (e.g. to orange). In this simple way, we are able to distinguish and separate genes that are not overlapping and are, therefore, differentially expressed.

However, from Fig. 1, it is important to note that gene expressions are variable even between replicates and this fact should also be considered when determining DE genes. Thus, we overlaid the replicate data with the between condition data as well, and choose the DE genes as the ones that do not overlap in all overlaid scatters. To determine DE genes between anchor time (e.g. *t* = 0) and target time (e.g. *t* = 10 min) for *E. coli* and *S. cerevisiae*, and between untreated and ETO treated mouse ESC cells, we overlaid the anchor time (or untreated) and target time (or treated) replicate data together onto the required axes (Fig. 2b). As the 2 replicates for each of the two conditions resulted in 4 combinatory comparisons (replicate 1-condition 1 vs. replicate 1-condition 2, replicate 2-condition 1 vs. replicate 1-condition 2, and so on), we chose DE genes as those that do not overlap in all combinations. In other words, the genes from the two-condition scatter that do not overlap (green dots) are the actual DE genes, considering the replicate combinatorial variability. In this way, we can visualize and track DE genes more objectively for every time point or condition than setting an arbitrary expression threshold cut-off.

One limitation of this approach, however, is the size of dot used to represent a gene; a larger size will result in less DE genes compared to a smaller size used as there will be larger artificial overlap due to size on the scatter plots (Fig. S3). To overcome this, we performed scatter plot overlay for a range of dot size and computed noise (see above section) for the DE genes,  as well as for the remaining (non-DE) genes for each dot size used (Fig. 2c and Table 1). As expected, as the dot size increases, the number of DE genes decreased.

**Table 1:** Number of differentially expressed genes detected by ScatLay at various scatter dot sizes.

Scatter dot size	Number of DE genes in *E. coli*	Number of DE genes in *S. cerevisiae*	Number of DE genes in mouse ESC
0.001	3169	4455	7916
0.002	2216	3191	5019
0.003	1569	2483	3704
0.004	1194	2061	2932
0.005	975	1798	2445
0.006	832	1626	2091
0.007	741	1461	1807
0.008	679	1333	1576
0.009	622	1241	1410
0.01	582	1159	1243

To determine a more objective way to choose the correct dot size for selecting DE genes, we utilized the noise analysis again. As shown in Fig. 2a, the increased noise between conditions compared to replicates is due to DE genes, thus we used the average replicate noise threshold as a means to select the dot size (Fig. 2c). For *E. coli*, the size is 0.004 log_10_(TPM) which indicates 1194 DE genes while for *S. cerevisiae*, the indicated size is 0.001 log_10_(TPM) resulting in 4455 DE genes. For mouse ESC, dot size of 0.002 log_10_(TPM) yields 5019 DE genes. However, for simplicity, we used the most conservative dot size of *E. coli*, 0.004 log_10_(TPM), for all cell types. For this, we obtained 2061 and 2932 DE genes for *S. cerevisiae* and mouse ESC, respectively.

Particularly, when we evaluate the noise of the DE genes and the remaining non-DE genes, we find the latter’s noise similar to replicate noise levels and remarkably lower than DE genes’ noise (Fig. 2d). This confirms that our selected DE genes are responsible for increasing noise observed between time points. Note that the overlay of data is not restricted to replicate data, it can also be overlaid across multiple repeat datasets but with 2 replicates at a time. Figure S4 and Table S2 shows the triplicate data, available only for *E. coli*, is compared at all 3 possible combinations, and the total number of DE genes was almost the same (between 1191 and 1194).

### Correlation and PCA shows significant response of DE genes

Previously, we have used Pearson auto-correlation and principal component (PC) metrics to track the global, local and attractor gene expression responses of several cell types^13,21–23^. For studying Toll-like receptor induced immune response, the correlation metrics revealed that immune-related *local* genes were highly responsive while myriad *global* genes showed significantly less response^21,22^. In a similar way, for *E. coli*, we showed the subset of *attractor* genes, crucial for cell state transition, showed the most pronounced correlation metrics, while the rest of transcriptome lacked significance^23^. The PC metrics revealed that the *attractor* genes tracked almost identical trajectory compared with the transcriptome-wide response^23^. These data revealed that both correlation and PC metrics can be used to test the significance of Scatlay-derived DE genes.

**Figure 3:**
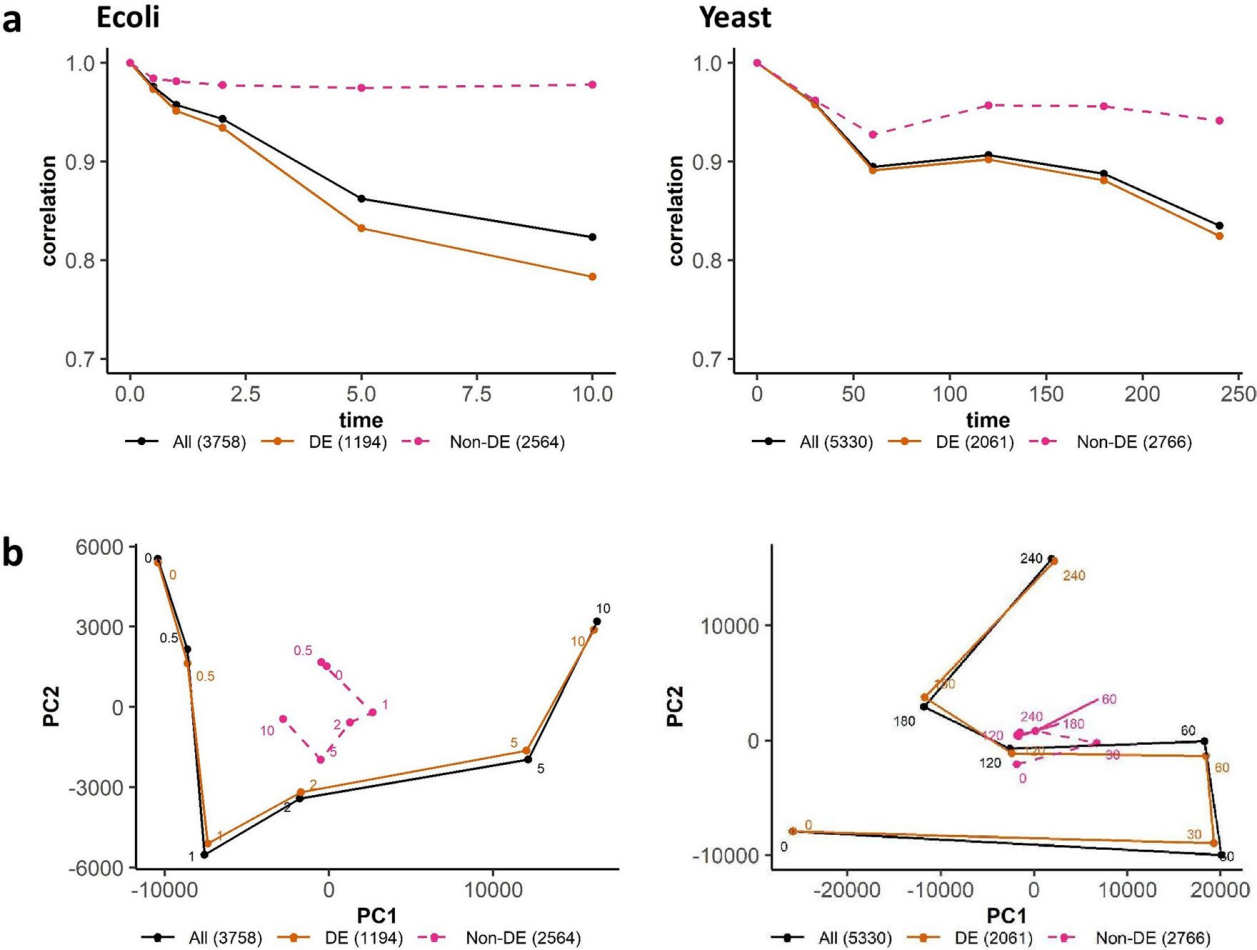
Auto-correlation and principal component (PC) analysis of whole transcriptome, DE genes and non-DE genes. (**a**) Pearson correlation and (**b**) Gene expression trajectory on PC1-PC2 space between time *t*_*0*_ (0 min) and *t*_*i*_ (0, 0.5, 1, 2, 5, 10 min for *E. coli* – left panels) and 0, 30, 60, 120, 180, 240 min for *S. cerevisiae* – right panel) of whole transcriptome (solid black), DE genes at scatter dot size 0.004 log(TPM) (solid orange), and non-DE genes (dashed pink). The PC trajectories were obtained by taking the average trajectories of 2 replicates. The first 2 PCs account for 91.76% total variance in *E. coli* and 80.25% in *S. cerevisiae*.

Here, we checked the progressive time response of (i) *whole transcriptome*, (ii) *DE genes*, (iii) *rest of transcriptome without DE genes (non-DE)*, using the same statistical metrics for *E. coli* and *S. cerevisiae* only, as the time-series data is not available for mouse (Fig. 3). Both auto-correlation and PC metrics reveal that the DE genes dominates transcriptome-wide response, while removing them (rest of transcriptome or non-DE) show highly subdued response. In other words, the ScatLay-derived DE genes are key for the progressive response of both cell types.

### Comparison of ScatLay with other DE gene methods

**Figure 4:**
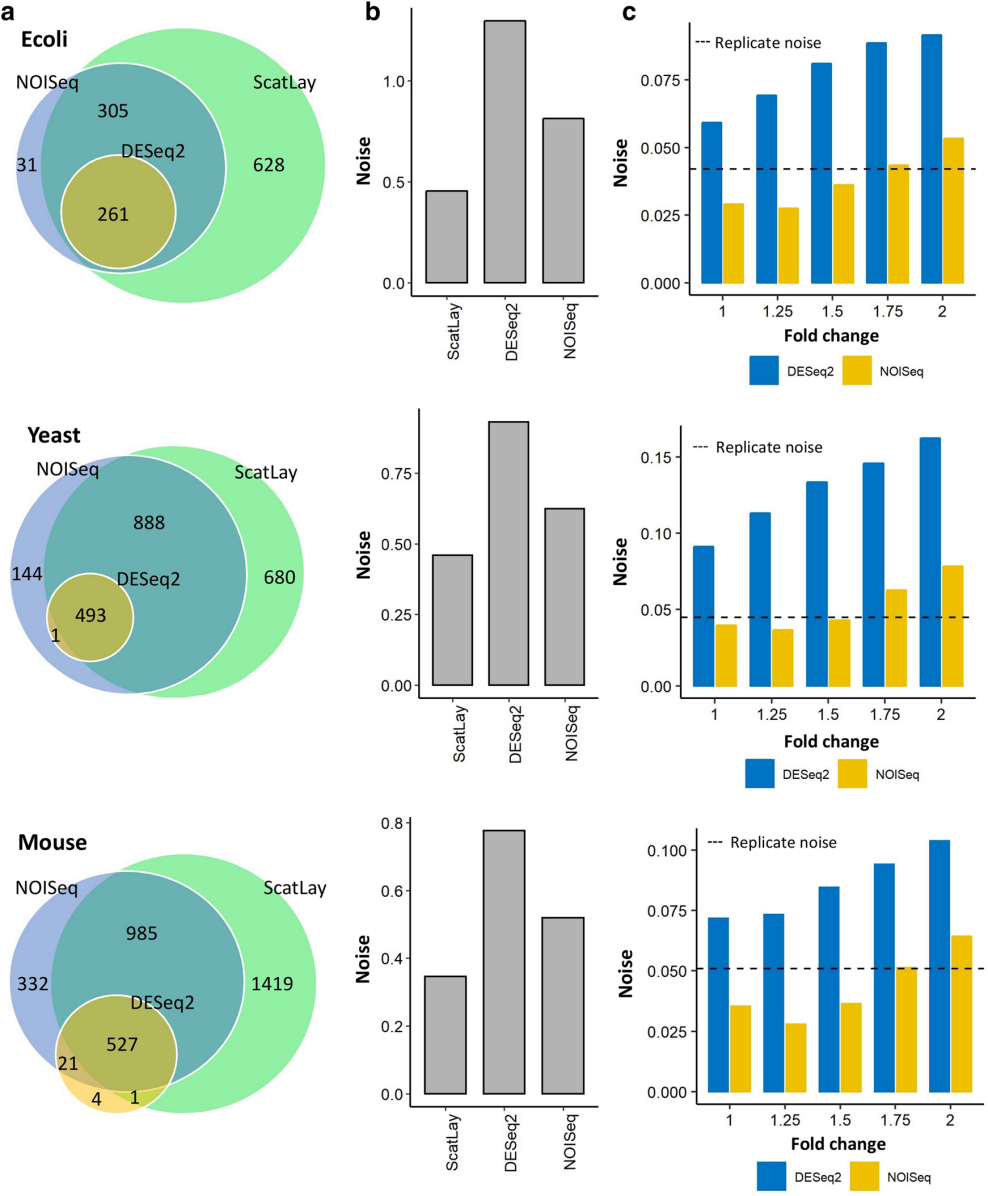
Comparison of DE genes by ScatLay, DESeq2, and NOISeq. (**a**) Number of DE genes detected by ScatLay at dot size 0.004 (green), DE genes detected by DESeq2 (dark yellow) and NOISeq (light purple) methods with expression fold change above 2 and adjusted *p *value below 0.05 for *E. coli* (top panel), *S. cerevisiae* (middle panel), and mouse ESC (bottom panel). (**b**) Expression noise due to the respective DE genes detected by ScatLay, DESeq2 and NOISeq. (**c**) Averaged expression noise between 2 time points due to the non-DE genes detected by DESeq2 (blue) and NOISeq (yellow) with expression fold-change threshold varying from 1.0 to 2.0, with adjusted *p* value below 0.05, in comparison to whole-transcriptome noise between replicates of the same condition (dashed line). Non-DE noise level by NOISeq method at 1.75-fold threshold for *E. coli* (top panel) and mouse ESC (bottom pabel), and 1.5-fold threshold for *S. cerevisiae* (middle panel) are similar to the replicate noise level. With *p* value cut-off at 0.05, DESeq2 non-DE genes show higher noise value than replicate noise at every fold change threshold. For DESeq2, *p*-value was raised to 0.25 to attain similar noise level of non-DE genes with replicate noise.

Next, we compared our results with other commonly used techniques based on DESeq2 and NOISeq methods with the conventional threshold of twofold expression changes and 0.05 *p* value cut-offs. Notably, ScatLay produces more DE genes than both DESeq2 (261 genes in *E. coli*, 494 genes in *S. cerevisiae* and 553 in mouse ESC) and NOISeq (597 genes in *E. coli*, 1526 genes in *S. cerevisiae* and 1865 in mouse ESC) (Fig. 4a). One of the reasons for this, based on our noise evaluation (Fig. 4b), is that both methods adopt arbitrary threshold cutoffs that are generally more conservative. The stringent thresholds applied on NOISeq and DESeq2 give rise to DE genes with higher noise level than ScatLay DE genes for all 3 cell types. In this case, our noise analysis could help determine a better threshold cutoff for higher coverage (Fig. 4c). For NOISeq, we observe that, with a *p* value cut-off at 0.05, expression fold threshold for *E. coli* and mouse ESC yields a value of 1.75, giving rise to 780 and 2705 DE genes, respectively, while it is 1.5 for *S. cerevisiae*, providing 2734 DE genes when matched with ScatLay noise benchmark.

For DESeq2, however, at any expression fold threshold cutoff above 1 with *p *value at 0.05 yields noise that are greater than ScatLay noise benchmark for all cell types*.* This indicate that DESeq2 is very stringent initially and our noise analysis could be used in conjunction to improve the overall coverage of DE genes. Thus, expression noise analysis is a useful tool to provide higher coverage of DE genes, and it can be used in conjunction with both ScatLay and other DE analysis methods like the popularly used DESeq2 or NOISeq, as discussed here.

To obtain a reduced or finer set of DE genes in ScatLay, we derived a method to determine a threshold cutoff based on *p* value estimation from kernel density estimation (Materials and Methods). To determine the probability whether a gene is DE, 2D kernel density estimation allows determining the possibility for a gene in the between-condition scatter to be overlapped by the between-replicate scatter (Fig. S5a). We applied the conventional *p* value cutoff of 0.05, in conjunction with ScatLay at scatter dot size 0.004 log_10_(TPM) (Fig. 2c), and found 815, 1744 and 2091 DE genes in *E. coli*, *S. cerevisiae,* and mouse ESC data, respectively. We also further included two fold expression threshold to ScatLay DE genes, and most of ScatLay-specific DE genes were eliminated by this criterion (Fig. S5b). For these 2 commonly used arbitrary cutoffs, ScatLay DE genes consist mainly of the overlapping DESeq2 and NOISeq DE genes. Notably, ScatLay still show higher coverage than NOISeq and DESeq2 DE genes. (Fig. S5b and Table 2).

**Table 2:** Number of differentially expressed genes detected by ScatLay with different cutoff criteria and their percentage coverage of DESeq2 and NOISeq DE genes.

	Number of genes	DESeq2 Coverage	NOISeq Coverage
***E. coli***			
ScatLay	1194	100%	100%
ScatLay with *p* value cutoff	815	100%	94.50%
ScatLay with *p* value and expression fold cutoff	563	100%	94.00%
NOISeq with *p* value and expression fold cutoff	597	100%	–
DESeq2 with *p* value and expression fold cutoff	261	–	43.70%
**Yeast**			
ScatLay	2061	100%	90.60%
ScatLay with *p* value cutoff	1744	99.80%	88.90%
ScatLay with *p* value and expression fold cutoff	1353	99.80%	88.50%
NOISeq with *p* value and expression fold cutoff	1526	100%	–
DESeq2 with *p* value and expression fold cutoff	494	–	32.30%
**Mouse**			
ScatLay	2392	95.50%	82.30%
ScatLay with *p* value cutoff	2091	95%	74.40%
ScatLay with *p* value and expression fold cutoff	1382	95%	73.70%
NOISeq with *p* value and expression fold cutoff	1865	99.10%	–
DESeq2 with *p* value and expression fold cutoff	553	–	29.40%

**Figure 5:**
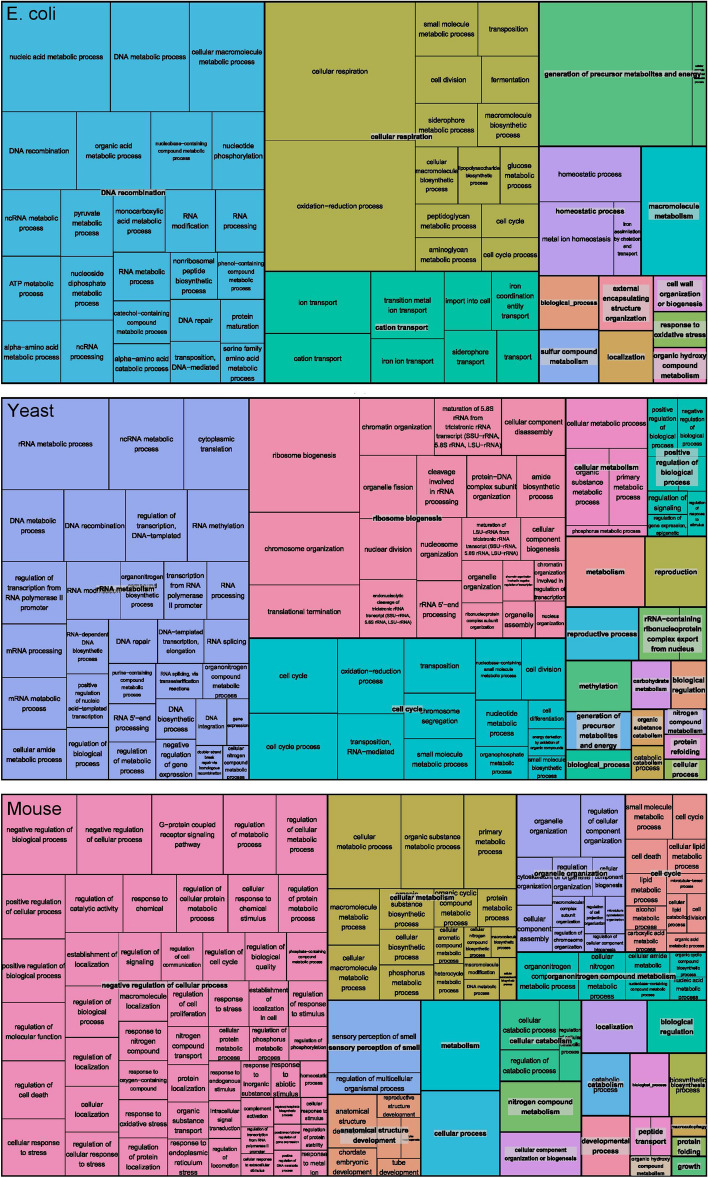
Enriched gene ontology of DE genes detected by ScatLay at scatter dot size 0.004 log10(TPM) and *p* value threshold at 0.05 for *E. coli* (top panel), *S. cerevisiae* (middle panel), and mouse ESC (bottom panel). Enrichment analysis were first retrieved from Gene Ontology Consortium with defaulted over-representation test parameters, and then refined and visualized by REVIGO tool^32^. The full list of enriched gene ontology terms is shown in Table S3.

Finally, we conducted gene enrichment analysis (Gene Ontology Consoritum^24^) on the DE genes detected by ScatLay with a *p* value threshold of 0.05. We observed that the 815 DE genes of *E. coli* in aerobiosis are mostly enriched in cellular respiration, DNA processes and homeostasis (Fig. 5 and Table S3). The 1744 DE genes of *S. cerevisiae* in hypoxia largely consist of RNA metabolism, ribosome biogenesis, and methylation, whereas, processes such as anatomical structure development, smell sensory perception, and cell cycle are elucidated for the 2091 DE genes of mouse ESC in ETO treatment (Fig. 5 and Table S3).

Notably, we observe that a small number of DE genes detected by NOISeq were not picked up by ScatLay at *p* value < 0.05 (34 genes in *E. coli*, 171 genes in *S. cerevisiae* and 478 genes in mouse ESC—Fig. S5b, top panel). Nevertheless, gene enrichment analysis did not show any known biological function for these NOISeq-specific DE genes from *E. coli* and *S. cerevisiae* types. For mouse ESC, the 478 NOISeq-specific genes are enriched in 9 biological processes only, consisting of mostly regulation of cellular process and phosphorous metabolism (Table S4). On the other hand, the 252 ScatLay-specific DE genes (*p* value < 0.05) in *E. coli* show enrichment in serine amino acid metabolism, locomotion, and translation processes. In *S. cerevisiae,* the 389 ScatLay-specific genes are enriched in 128 biological processes, including ribosome biogenesis, translation, and gluconeogenesis. In mouse ESC, 430 enriched biological processes are detected for the 704 ScatLay-specific genes, such as developmental process, cell cycle phase transition, and regulation of apoptosis (Table S5).

Overall, ScatLay elucidates statistically reliable DE genes with overall higher coverage, without or with threshold cutoff, as compared with DESeq2 and NOISeq. As the 3 methods compared originate from distinct statistical methodologies and assumptions, it is inevitable to obtain a small number of distinct DE genes pertaining to each method. Notably, even with *p* value and expression threshold cutoff, ScatLay covered almost all the genes of DESeq2. However, NOISeq picks up several distinct DE genes not captured by ScatLay. Nevertheless, further experimental work is necessary to investigate these distinct DE genes captured by each method.

## Conclusion

Here, we developed a new method, implemented in R programming language with a graphical user interface, to identify and visualize DE genes through overlaying transcriptome-wide expressions between samples (replicates, condition or time points). Unlike approaches that uses arbitrary threshold levels to select DE genes, here the genes are checked for replicate variability before sample variability by our noise analyses. Overall, our method provides a novel way to uncover DE genes that are not biased by user defined threshold cutoff and are able to produce a larger overall coverage. Nevertheless, we also provide an optional utilization of *p* value cut-off, derived from 2D kernel density of between-replicate scatter plots, if further reduction of DE genes is required, for example, to focus only on the highly variable genes.

## Materials and methods

### Data

We obtained time-series RNA-Seq dataset, in raw read counts, for *Escherichia coli* in aerobiosis (GEO accession number GSE71562)^25^, *Saccharomyces cerevisiae* in hypoxia (GEO accession number GSE85595)^26^, and *Mus Musculus* embryonic stem cell in different treatment or gene knock-out conditions (GEO accession number GSE137138)^27^.

Briefly, for the *E. coli*, K-12 strain W3110 was grown in a 3-L continuously stirred tank bioreactor anaerobically at pH7 and 37 °C. The first sample was drawn (*t* = 0) when OD of 3 at 600 nm was achieved, and air supply of 1L/min was then initiated. Subsequent samples were taken, at *t* = 0.5, 1, 2, 5 and 10 min^25^.

For *S. cerevisiae* (strain yMH914 with wildtype HAP1), cells were subjected to 100% nitrogen gas and collected after 0, 5, 10, 30, 60, 120, 180, and 240 min^26^. Total RNA was extracted and mRNAs were enriched by polyA selection.

Mouse ESCs were derived from blastocysts of 2–6-month-old male mice from C57BL/6 strain. Mouse ESCs from E14tg2a cell lines were cultured in 2i/LIF medium, and treated with H_2_O (control), or Etomoxir (ETO), or then released from ETO for another 4 days (ETO-released). Wild-type mouse ESCs and Mof-deleted (Mof knock-out or Mof-KO) mouse ESCs were cultured in 2i/LIF medium with Ethanol (WT) or 4-OHT (Mof-KO)^27^. In this study, we selected only the control and ETO conditions for DE analysis.

In all datasets, the cDNAs were prepared into a sequencing library, multiplexed and sequenced by an Illumina HiSeq 2500 sequencer. In total, there were 4240, 6494 and 17,392 non-zero gene expressions with gene lengths for *E. coli*, *S. cerevisiae* and mouse ESC, respectively. For our analysis, we chose replicate data with best pairwise correlation for each species at each time point.

### Statistical distributions fitting

Fitting gene expression distributions was performed using the Maximum-likelihood Fitting method (fitdistplus packge^28^ for parameter fitting and the mass package^29^ for log-normal, Pareto, Burr, Loglogistic, Weibull and Burr distributions^30^).

### Gene expression noise

Gene expression noise, *η*^2^, is defined by gene expression variance (*σ*^2^) over square of mean (*μ*^2^)^8,10,16^. To compute transcriptome-wide noise, we need to first evaluate noise of each gene (*i* = 1, …, *m*) between pairs of replicates or samples (*j*,*k* = 1,…,*n*):

$${\eta }_{i\left(jk\right)}^{2}=\frac{{\sigma }_{i\left(jk\right)}^{2}}{{\mu }_{i\left(jk\right)}^{2}}=2\frac{{\left({x}_{ij}-{x}_{ik}\right)}^{2}}{{\left({x}_{ij}+{x}_{ik}\right)}^{2}}$$

where *x*_*ij*_ and *x*_*ik*_ is the expression value of the *i*th gene in the *j*th and *k*th replicates/samples, and ${\sigma }_{i\left(jk\right)}^{2}={({x}_{ij}-{x}_{ik})}^{2}/2$ and ${\mu }_{i\left(jk\right)}^{2}={({x}_{ij}+{x}_{ik})}^{2}/4$ are the variance and square mean expression. We then summed the noise values of all genes between pairs of samples (*j,k* = *1,…,n*) to calculate the total noise for each transcriptome, such as

$${\eta }^{2}=\sum_{i=1}^{m}{\eta }_{i}^{2}$$

where *m* is the total number of genes.

### Probability of differential expression for Scatlay

We select DE genes from the between-condition scatter as those not overlapped onto the between-replicate scatters. Thus, the probability whether a gene is differentially expressed equates the probability for its between-condition gene expression vector [${x}_{i1, } {x}_{i2}$] (with *i* = 1, …, *m*) to fall into the cloud of gene expression scatter between 2 replicates:

$$p= {\int }_{-\infty }^{({x}_{i1}, {x}_{i2})}f({x}_{i1},{x}_{i2})$$

in which *f* is the estimated kernel density function on between-replicate scatters:

$$f\left({x}_{i1},{x}_{i2}\right)=\frac{1}{2}({G}_{H}\left({x}_{i1}-{X}_{1}\right)+{G}_{H}\left({x}_{i2}-{X}_{2}\right))$$

where ${X}_{k}$ is the concatenated gene expression vector in 2 conditions at replicate *k* (*k* = 1,2), ${G}_{H}$ is 2D Gaussian kernel function at variance matrix (bandwidth) *H*, and the variance matrix *H* was estimated based on ${X}_{1}$ and ${X}_{2}$ vectors using *hpi* function from *ks* package^31^.

## Supplementary information

Supplementary Information.

Supplementary Table 3.

Supplementary Table 5.

## Data Availability

The ScatLay source code with user instructions can be found on URL: https://github.com/buithuytien/ScatLay.
