# Supplementary material for: ScatLay: utilizing transcriptome-wide noise for identifying and visualizing differentially expressed genes
Source: Sci Rep. 2020 Oct 15;10:17483. doi: 10.1038/s41598-020-74564-1 (PMC7566603; doi:10.1038/s41598-020-74564-1)
Supplement: Supplementary file 1 — Supplementary Information. [file 41598_2020_74564_MOESM1_ESM.pdf]

Supplementary Figures and Tables

ScatLay: Utilizing Transcriptome-wide Noise for Identifying and Visualizing Differentially Expressed Genes

Thuy Tien Bui[1], Daniel Lee[2], and Kumar Selvarajoo[1,3,*]

[1]Singapore Institute for Food and Biotechnology Innovation, Agency for Science, Technology & Research (A*STAR), 61 Biopolis Drive, Singapore 138673.

[2]School of Computer Science and Engineering, Nanyang Technological University, 50 Nanyang Avenue, Singapore 639798.

[3]Synthetic Biology for Clinical and Technological Innovation (SynCTI), National University of Singapore, 28 Medical Drive, Singapore 117456

*Corresponding author email: kumar_selvarajoo@sifbi.a-star.edu.sg

Supplemental figure legend

Figure S1: **Comparing transcriptome-wide data with statistical distributions**. Cumulative distribution functions and Quantile-quantile plot in *E. coli* (**a** and **b**), *S. cerevisiae* (**c** and **d**), and mouse ESC (**e** and **f**), between transcriptome data (black colour) and lognormal (red), Pareto (light green), Burr (cyan), log-logistic (blue), Weibull (purple), and gamma (grey) statistical distributions at t = 0, 0.5, 1, 2, 5, 10 minute for *E. coli*, t = 0, 5, 10, 30, 60, 120, 180, 240 minute for *S. cerevisiae*, and conditions (wild type, Mof KO, Control, ETO treated, ETO released) for mouse ESC. Genes with expression level above TPM = 5 for *E. coli* and TPM = 2 for *S. cerevisiae* and mouse ESC (vertical black dashed line) closely follow the best fit theoretical distribution, which is log-normal for *E. coli* and mouse ESC, and Burr or Log-logistic for *S. cerevisiae*.

Figure S2: Transcriptome-wide noise. Between 2 replicates at *t* = 0, 0.5, 1, 2, 5, 10 min for *E. coli* (left panel), *t* = 0, 5,10, 30, 60, 120, 180, 240 min for *S. cerevisiae* (right panel), and between conditions (wild type, Mof KO, Control, ETO treated, ETO released) for mouse ESC, after removing lowly expressed genes (Fig. S1).

Figure S3: **Demonstration of dot size effect on determining DE genes.** Dot size of 0.0125 log10(TPM), resulted in 482 DE genes (left panel), while dot size of 0.004 log10(TPM), resulted in 1,194 DE genes (right panel) for *E. coli*, used as an example.

Figure S4: Expression noise as an indicator of differential transcriptional mechanism in 3 pairwise combinations of *E. coli* data. a) Expression noise between 2 replicates of E. coli at the same time point (first 6 bars from the left), and between anchor and target time points (the remaining 9 bars on the right) in 15 combinations of 3 replicates and 2 conditions. The r_* annotation denotes replicate name (r1, r2, r3), and the t_* annotation denotes time point, in which t = 0 corresponds to time point 1 (t1) and t = 10 minutes correspond to time point 2 (t2). b) Expression noise between anchor and target time points due to DE genes (filled circle) and non-DE genes (filled triangle) with scatter dot size ranging from 0.001 to 0.01 log10(TPM). Scatter dot size at 0.004 resulted in non-DE gene set whose expression noise between anchor and target time points is comparable to the averaged whole-transcriptome noise between 2 replicates (dashed blue line) at all 3 pairs. There are 1,194 DE genes when using replicates 1 and 2 (left panel - this is the data shown in original manuscript), 1,191 DE genes with replicates 1 and 3 (middle panel), and 1,193 DE genes with replicates 2 and 3 (right panel).

Figure S5: 2D Kernel density – based significance testing for ScatLay. **a)** 2D Gaussian Kernel Density estimated from the between-replicate scatters of *E. coli* for any two conditions (as a representative for all data). *P*-value obtained for each gene through the integration of estimated density from –Inf to the between-condition scatter coordinate of that gene. **b)** Number of DE genes detected by ScatLay (green) at scatter dot size 0.004 (Fig. 2c), with KDE-based *p*-value cutoff at 0.05 (top panel), and 2-fold expression together with 0.05 *p*-value cutoff (bottom panel), and DE genes detected by DESeq2 (dark yellow) and NOISeq (light purple) with expression fold change above 2 and adjusted p-value below 0.05 in E. coli (left panel), S. cerevisiae (middle panel), and mouse ESC (right panel).

Figure S6: **Screenshots from ScatLay desktop application.** Top-left panel: Reading gene expression matrix in either raw read count format, or normalized (such as TPM, RPKM or TMM units) format. Top-right panel: Pre-processing gene expression data, with expression normalization if raw read count was inputted, and removing lowly expressed genes. Bottom–left panel: Overlaying between-condition and between-replicate scatter plots. User change scatter dot size and *p*-value threshold to adjust specificity of DE genes. The non-overlapping genes with *p*-value satisfying threshold level are highlighted in green at the bottom right scatter. Bottom-right panel: List of DE genes with their associating *p*-value. User can download this set of gene as comma separated values (.csv) file.

E.coli

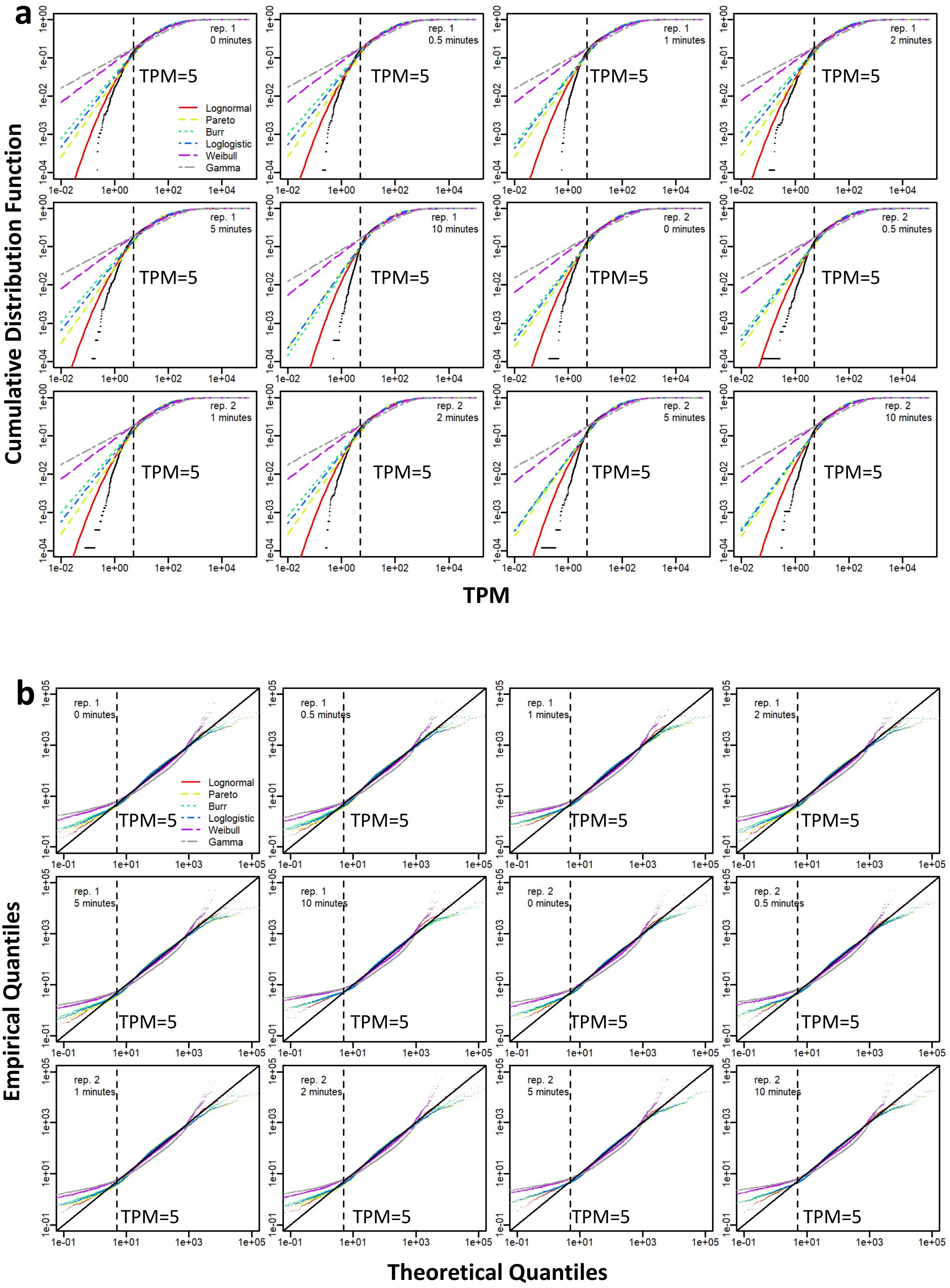

Figure S1

Yeast

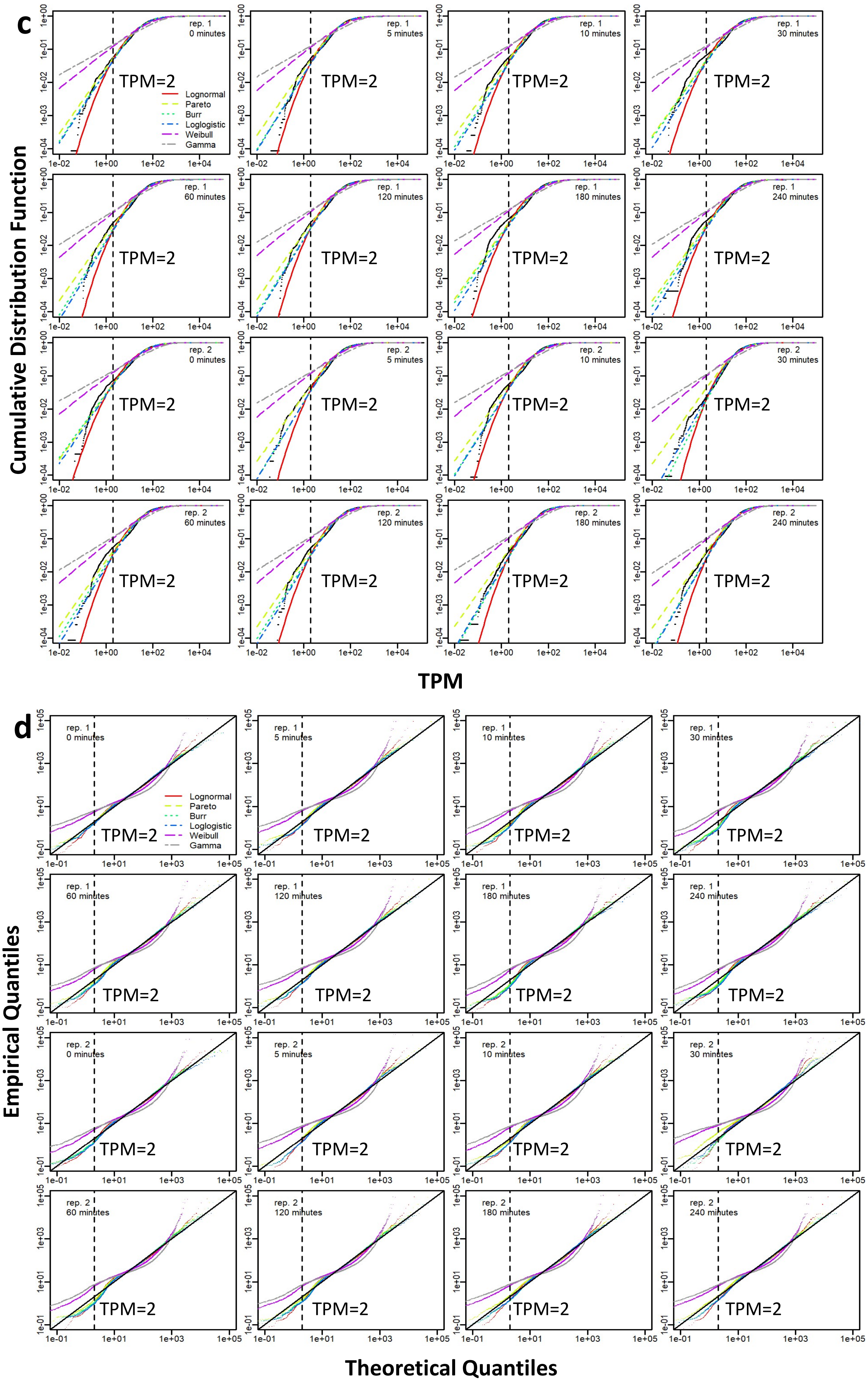

Figure S1 cont.

Mouse

e

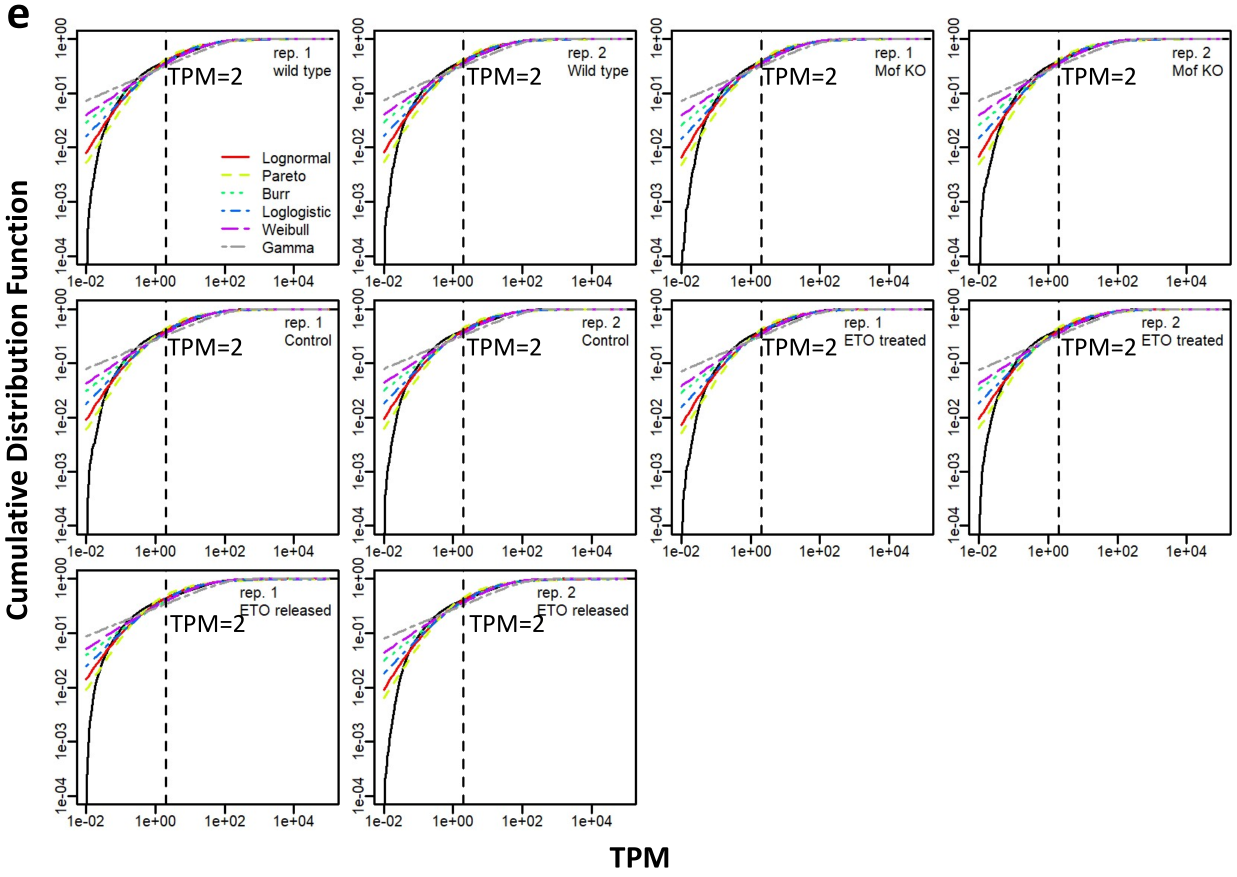

f

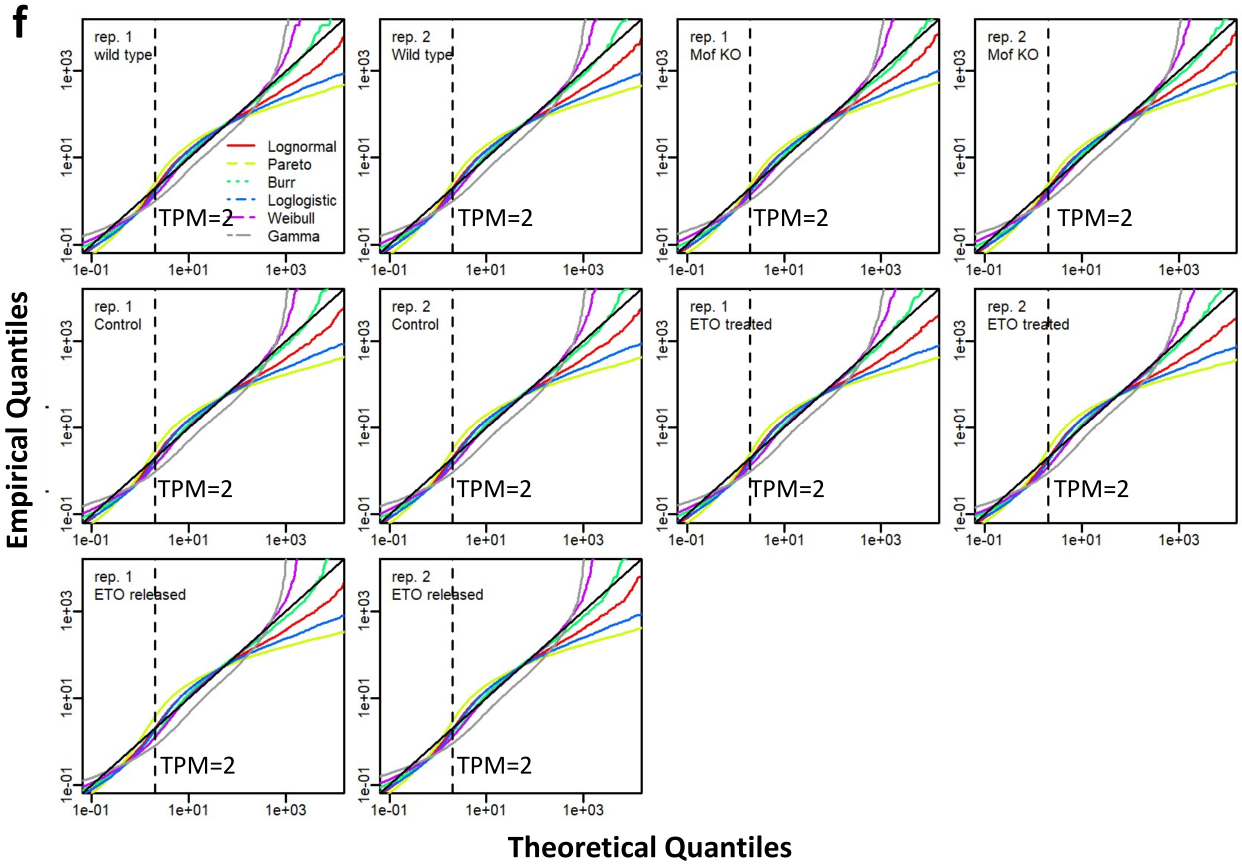

Figure S1 cont.

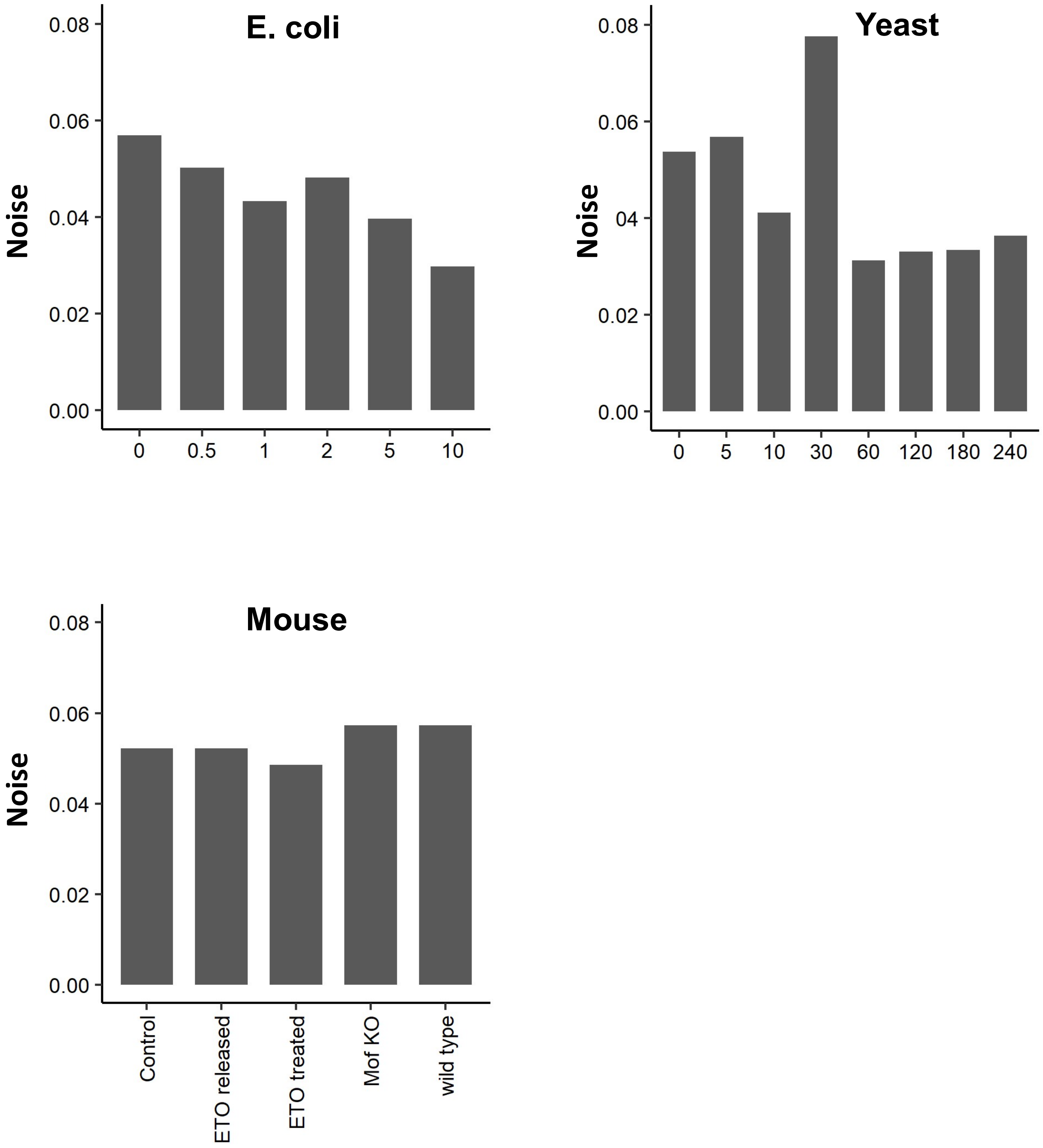
E. coli
Yeast
Mouse
Noise
0.00
0.02
0.04
0.06
0.08
04
0
0.5
1
2
5
10
30
60
120
180
240
Control
ETO released
ETO treated
Mof KO
wild type

Figure S2

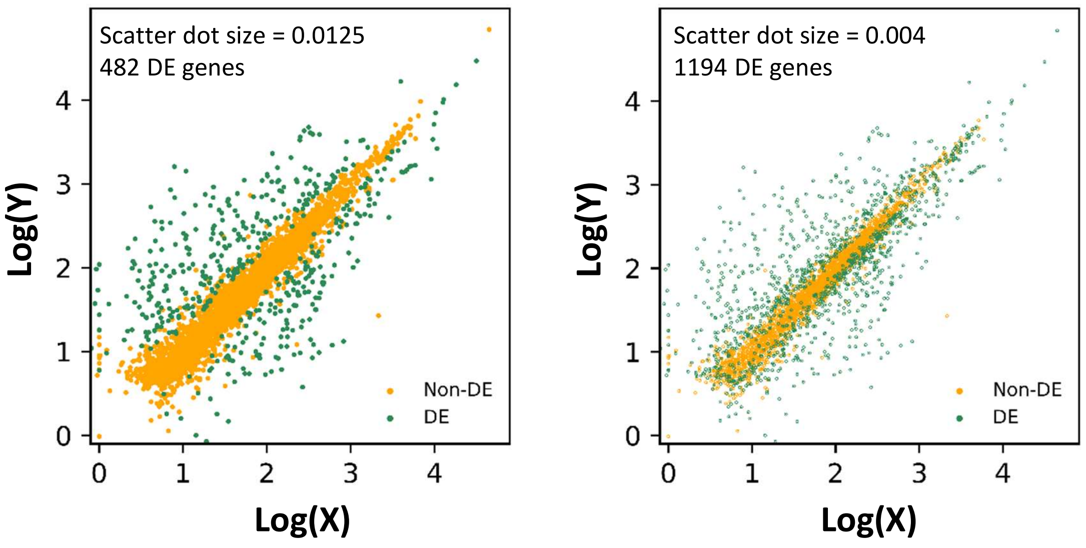
Scatter dot size = 0.0125
482 DE genes
Non-DE
DE
Log(Y)
Log(X)
Scatter dot size = 0.004
1194 DE genes
Non-DE
DE
Log(Y)
Log(X)

Figure S3

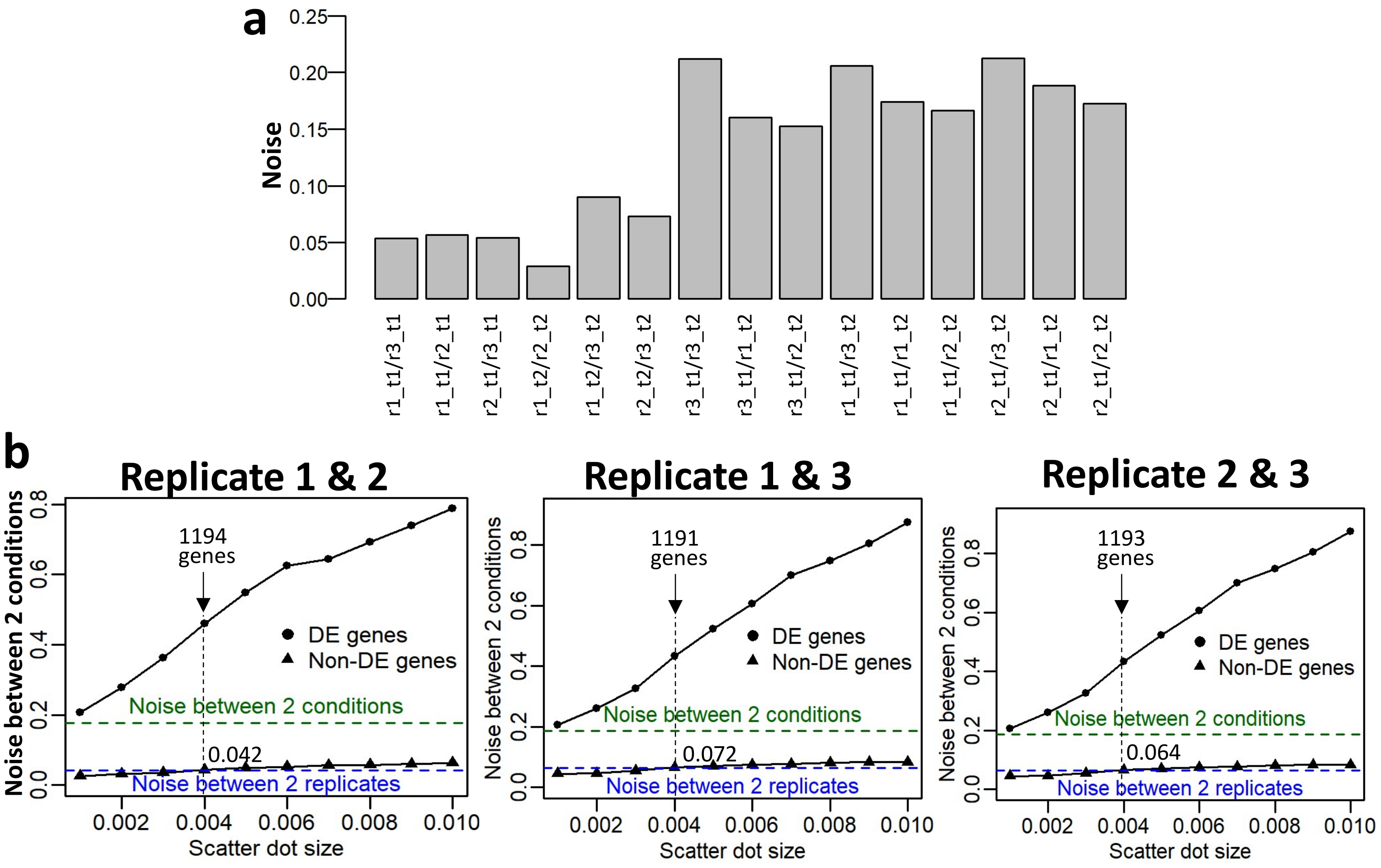
a
Noise
0.25
0.20
0.15
0.10
0.05
0.00
r1_t1/r3_t1
r1_t1/r2_t1
r2_t1/r3_t1
r1_t2/r2_t2
r1_t2/r3_t2
r2_t2/r3_t2
r3_t1/r3_t2
r3_t1/r1_t2
r3_t1/r2_t2
r1_t1/r3_t2
r1_t1/r1_t2
r1_t1/r2_t2
r2_t1/r3_t2
r2_t1/r1_t2
r2_t1/r2_t2
b
Replicate 1 & 2
Replicate 1 & 3
Replicate 2 & 3
Noise between 2 conditions
1194 genes
1191 genes
1193 genes
DE genes
Non-DE genes
Noise between 2 conditions
Noise between 2 replicates
0.042
0.072
0.064
0.0
0.2
0.4
0.6
0.8
0.002
0.004
0.006
0.008
0.010
Scatter dot size

Figure S4

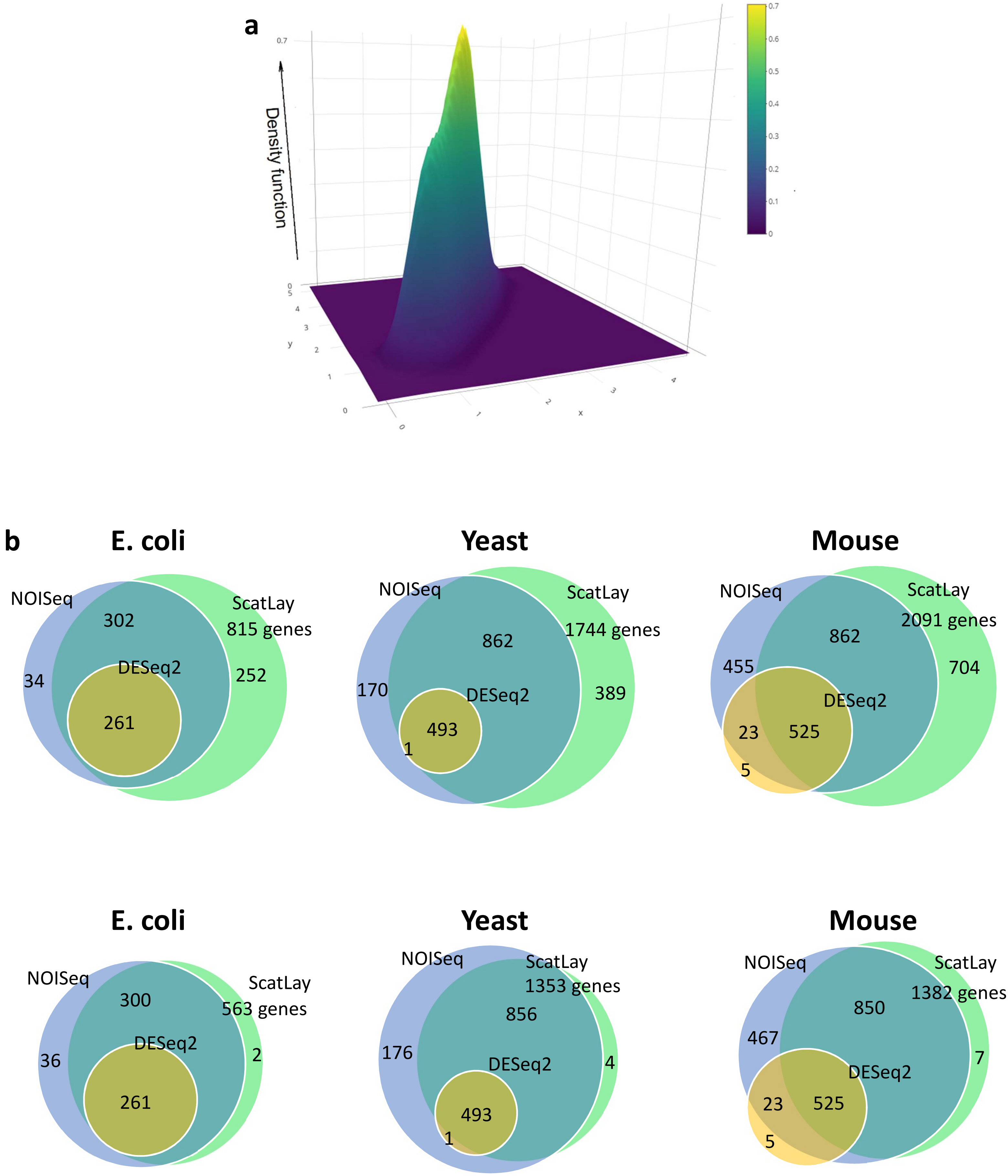
a
Density function
x
y
b
E. coli
NOISeq
ScatLay
815 genes
302
34
DESeq2
252
261
Yeast
NOISeq
ScatLay
1744 genes
862
170
DESeq2
389
493
1
Mouse
NOISeq
ScatLay
2091 genes
862
455
704
DESeq2
23
525
5
E. coli
NOISeq
ScatLay
563 genes
300
36
DESeq2
2
261
Yeast
NOISeq
ScatLay
1353 genes
856
176
DESeq2
4
493
1
Mouse
NOISeq
ScatLay
1382 genes
850
467
7
DESeq2
23
525
5

Figure S5

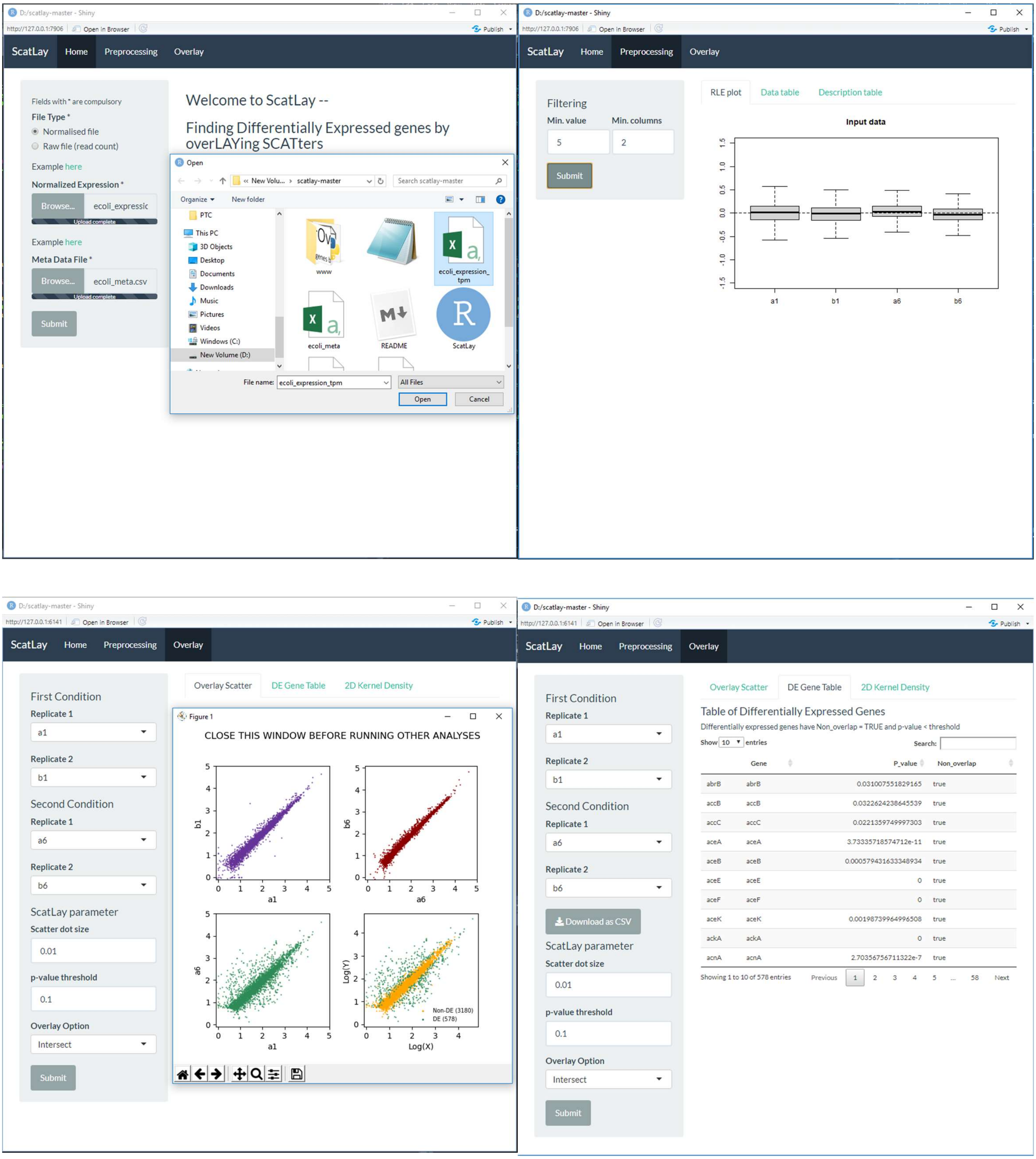
D:/scatlay-master - Shiny
ScatLay
Home
Preprocessing
Overlay
Fields with * are compulsory
File Type *
Normalised file
Raw file (read count)
Example here
Normalized Expression *
Browse...
Upload complete
Meta Data File *
ecoli_meta.csv
Submit
Welcome to ScatLay --
Finding Differentially Expressed genes by overLAYing SCATters
Open
Organize
New folder
This PC
3D Objects
Desktop
Documents
Downloads
Music
Pictures
Videos
Windows (C:)
New Volume (D:)
www
ecoli_expression_tpm
ecoli_meta
README
ScatLay
File name:
All Files
Open
Cancel
Filtering
Min. value
Min. columns
RLE plot
Data table
Description table
Input data
First Condition
Replicate 1
Replicate 2
Second Condition
ScatLay parameter
Scatter dot size
p-value threshold
Overlay Option
Intersect
Overlay Scatter
DE Gene Table
2D Kernel Density
Figure 1
CLOSE THIS WINDOW BEFORE RUNNING OTHER ANALYSES
Non-DE (3180)
DE (578)
Log(X)
Log(Y)
Download as CSV
Table of Differentially Expressed Genes
Differentially expressed genes have Non_overlap = TRUE and p-value < threshold
Show 10 entries
Search:
Gene
P_value
Non_overlap
Showing 1 to 10 of 578 entries
Previous
Next

Figure S6

Supplemental table legend

Table S1: Akaike's information criterion showing the best fitted statistical distribution to the transcriptome-wide expression of *E. coli*, *S. cerevisiae* and mouse ESC data.

Table S2: Number of DE genes detected in each pair of replicates in *E. coli* data (namely 1 and 2, 1 and 3, 2 and 3) by ScatLay at optimal scatter dots size (0.004), by DESeq2 with fold-change >= 2 and p-value < 0.05, and by NOISeq with fold-change >= 2 and p-value < 0.05.

Table S3: Enriched biological processes of DE genes detected by ScatLay with *p*-value < 0.05 for *E. coli* (815 genes), *S. cerevisiae* (1744 genes), and mouse ESC (2091 genes) data at scatter dot size 0.004 $\log_{10}$(TPM). The number of uniquely mapped gene IDs are 769, 1535 and 1802 genes for E. coli, Yeast, and Mouse respectively. Enrichment analysis was retrieved from Gene Ontology Consortium with defaulted over-representation test parameters.

Table S4: Nine enriched biological processes of DE genes detected by NOISeq but not picked up by ScatLay (478 NOISeq genes) in mouse ESC data. The number uniquely mapped gene is 345 genes. Enrichment analysis were retrieved from Gene Ontology Consortium with defaulted over-representation test parameters

Table S5: Enriched biological processes of DE genes specifically detected by ScatLay with *p*-value < 0.05 for *E. coli* (252 genes), *S. cerevisiae* (389 genes), and mouse ESC (704 genes) data. The number of uniquely mapped gene IDs are 232, 363 and 520 genes for E. coli, Yeast, and Mouse respectively. Enrichment analysis was retrieved from Gene Ontology Consortium with defaulted over-representation test parameters.

Table S1

E. coli

Time (minute)	Replicate	Lognormal	Pareto	Burr	Loglogistic	Weibull	Gamma	min_AIC
0	Rep. 1	48642.39	48836.06	48789.4	48803.99	49292.29	50337.54	Lognormal
0.5	Rep. 1	48491.43	48704.59	48633.85	48657.3	49097.76	50127.62	Lognormal
1	Rep. 1	48841.43	49051.21	49033.92	49034.86	49603.09	50638.59	Lognormal
2	Rep. 1	48526.78	48750.51	48671.86	48693.55	49125.86	50163.96	Lognormal
5	Rep. 1	48473.24	48706.49	48642.12	48656.2	49117.31	50172.92	Lognormal
10	Rep. 1	49269.51	49441.38	49439.1	49443.25	50215.4	51289.65	Lognormal
0	Rep. 2	48257.48	48423.97	48409.13	48412.21	49017.74	50139.47	Lognormal
0.5	Rep. 2	48983.88	49168.25	49156.31	49157.84	49753.5	50846.05	Lognormal
1	Rep. 2	48741.22	48971.46	48909.27	48926.51	49384.77	50424.94	Lognormal
2	Rep. 2	48488.08	48693.12	48650.92	48661.23	49186.1	50327.67	Lognormal
5	Rep. 2	48799.45	48976.71	48976.61	48974.65	49636.51	50734.56	Lognormal
10	Rep. 2	48829.55	49006.02	49002	49000.89	49646.14	50797.74	Lognormal

S. cerevisiae

Time (minute)	Replicate	Lognormal	Pareto	Burr	Loglogistic	Weibull	Gamma	min_AIC
0	Rep. 1	62251.87	62046.32	6203.294	62034.3	63465.49	65685.38	Burr
5	Rep. 1	62872.81	62632.09	62590.54	62591.02	64205.38	66426.87	Burr
10	Rep. 1	63624	63291.17	63265.71	63268.08	64822.66	67008.98	Burr
30	Rep. 1	65175.98	64741.05	64740.89	64778.81	66052.07	68147.86	Burr
60	Rep. 1	64851	64549.36	64509.83	64516.01	66002.93	67995.11	Burr
120	Rep. 1	64092.4	63844.22	63804.8	63804.95	65340.85	67466.85	Burr
180	Rep. 1	65258.36	64864.59	64863.5	64888.5	66187.8	68272.38	Burr
240	Rep. 1	65705.9	65356.69	65351.85	65377.61	66621.55	68445.76	Burr
0	Rep. 2	63616.34	63370.84	63370.80	63385.55	64591.03	66599.47	Burr
5	Rep. 2	62749.62	62443.72	62381.8	62379.8	64098.16	66455.41	Loglogistic
10	Rep. 2	62819.05	62523.05	62485.55	62484.28	64112.58	66440.08	Loglogistic
30	Rep. 2	61554.74	61458	61197.6	61223.92	63293.81	65545.35	Burr
60	Rep. 2	65323.74	64937.24	64916.44	64931.27	66342.51	68302.57	Burr
120	Rep. 2	64800.66	64490.71	64462.45	64469.13	65906.13	67880.98	Burr
180	Rep. 2	63396.9	63183.05	63074.05	63074.83	64813.86	66926.02	Burr
240	Rep. 2	64329.56	64094.88	64019.77	64019.96	65561.05	67444.42	Burr

Mouse embryonic stem cell

	Lognormal	Pareto	Burr	Loglogistic	Weibull	Gamma	min_AIC
WT.1	152626.1	156204.4	153044.6	154001.1	153550.6	159878.5	Lognormal
WT.2	151632	155211.9	152058.6	153012.3	152577.2	159197.9	Lognormal
Mof.KO.1	148181	151290.4	148757.1	149472.1	149436.7	156196.7	Lognormal
Mof.KO.2	148297.5	151459.9	148848.6	149587.8	149515.2	156301.1	Lognormal
Control.1	146187	149672.5	146711.8	147591.7	147239.9	154345.1	Lognormal
Control.2	147299.4	150802.5	147865.7	148725.7	148395.9	155388.8	Lognormal
ETO.1	157486.4	161122.5	157942.1	158998.1	158364.6	165125.3	Lognormal
ETO.2	158984.3	162839.1	159541.6	160624.5	159907.1	166697.2	Lognormal
Released.1	153600.9	157415	154453.4	155286.8	154914.3	162128.2	Lognormal
Released.2	151166.8	154591	151911.3	152690.2	152481.1	159772.6	Lognormal

Table S2

	ScatLay	DESeq2	NOISeq
Replicates 1 & 2	1194	261	597
Replicates 1 & 3	1191	239	530
Replicates 2 & 3	1193	262	627
Variability	0.25%	9.6%	18.1%

Table S4

GO biological process complete	Reference (22265)	Mouse ESC (345)	Mouse ESC (expected)	Over/under	Fold enrichment	Raw P-value	FDR
phosphorus metabolic process (GO:0006793)	1713	51	26.54	+	1.92	1.01E-05	1.46E-02
phosphate-containing compound metabolic process (GO:0006796)	1691	50	26.2	+	1.91	1.47E-05	1.93E-02
negative regulation of cellular metabolic process (GO:0031324)	2500	71	38.74	+	1.83	7.90E-07	1.78E-03
regulation of signal transduction (GO:0009966)	2856	73	44.25	+	1.65	2.24E-05	2.53E-02
negative regulation of cellular process (GO:0048523)	4747	117	73.56	+	1.59	9.76E-08	5.14E-04
negative regulation of biological process (GO:0048519)	5213	122	80.78	+	1.51	7.26E-07	1.91E-03
regulation of response to stimulus (GO:0048583)	3997	92	61.93	+	1.49	7.22E-05	4.57E-02
positive regulation of cellular process (GO:0048522)	5722	124	88.66	+	1.4	3.07E-05	2.85E-02
regulation of cellular process (GO:0050794)	11169	211	173.07	+	1.22	5.60E-05	4.02E-02
